# Association of systemic inflammation with shock severity, 30-day mortality, and therapy response in patients with cardiogenic shock

**DOI:** 10.1007/s00392-023-02336-8

**Published:** 2023-11-20

**Authors:** Angela Dettling, Jessica Weimann, Jonas Sundermeyer, Benedikt N. Beer, Lisa Besch, Peter M. Becher, Fabian J. Brunner, Stefan Kluge, Paulus Kirchhof, Stefan Blankenberg, Dirk Westermann, Benedikt Schrage

**Affiliations:** 1 Department of Cardiology, University Medical Center Hamburg-Eppendorf, Martinistr. 52, 20246 Hamburg, Germany; 2 German Center for Cardiovascular Research (DZHK), Partner Site Hamburg/Kiel/Lübeck, Hamburg, Germany; 3 Department of Intensive Care Medicine, University Medical Center Hamburg-Eppendorf, Hamburg, Germany; 4 Institute of Cardiovascular Sciences, University of Birmingham, Birmingham, UK; 5 Department of Cardiology and Angiology I, University Heart Center Freiburg, Bad Krozingen, Germany

**Keywords:** Cardiogenic shock, Systemic inflammation, Mechanical circulatory support, Mortality

## Abstract

**Background:**

Mortality in cardiogenic shock (CS) remains high even when mechanical circulatory support (MCS) restores adequate circulation. To detect a potential contribution of systemic inflammation to shock severity, this study determined associations between C-reactive protein (CRP) concentrations and outcomes in patients with CS.

**Methods:**

Unselected, consecutive patients with CS and CRP measurements treated at a single large cardiovascular center between 2009 and 2019 were analyzed. Adjusted regression models were fitted to evaluate the association of CRP with shock severity, 30-day in-hospital mortality and treatment response to MCS.

**Results:**

The analysis included 1116 patients [median age: 70 (IQR 58–79) years, 795 (71.3%) male, lactate 4.6 (IQR 2.2–9.5) mmol/l, CRP 17 (IQR 5–71) mg/l]. The cause of CS was acute myocardial infarction in 530 (48%) patients, 648 (58%) patients presented with cardiac arrest. Plasma CRP concentrations were equally distributed across shock severities (SCAI stage B–E). Higher CRP concentrations were associated with 30-day in-hospital mortality (8% relative risk increase per 50 mg/l increase in CRP, range 3–13%; *p* < 0.001), even after adjustment for CS severity and other potential confounders. Higher CRP concentrations were only associated with higher mortality in patients not treated with MCS [hazard ratio (HR) for CRP > median 1.50; 95%-CI 1.21–1.86; *p* < 0.001], but not in those treated with MCS (HR for CRP > median 0.92; 95%-CI 0.67–1.26; *p* = 0.59; *p*-interaction = 0.01).

**Conclusion:**

Elevated CRP concentrations are associated with increased 30-day in-hospital mortality in unselected patients with cardiogenic shock. The use of mechanical circulatory support attenuates this association.

**Graphical abstract:**

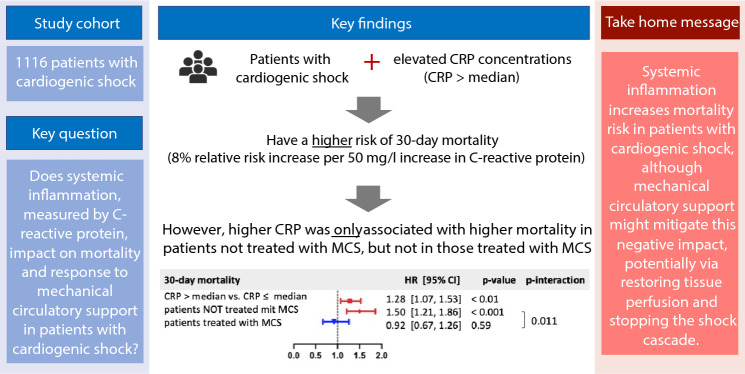

## Introduction

Cardiogenic shock (CS) remains a clinical challenge with a high mortality despite significant advances in overall cardiovascular medicine [1, 2]. The high mortality of CS likely reflects its complex and incompletely understood pathophysiology involving hemodynamic and circulatory disturbances, triggering systemic inflammation and organ dysfunction [3]. The degree of CS severity can be graded using the Society for Cardiovascular Angiography and Intervention (SCAI) shock classification and is associated with mortality in patients with CS [4, 5].

Only early percutaneous coronary intervention (PCI) of the infarct-related artery has been shown to improve survival in patients with CS caused by acute myocardial infarction (AMI), but the associated mortality risk remains high [6, 7]. Current hopes have been set in mechanical circulatory support devices (MCS) as a bridge to recovery, restoring cardiac output and tissue perfusion; yet the evidence on this is far from definitive and they come at its own cost of more complications [8–11].

Non-cardiovascular factors influence both, prognosis and response to treatment in patients with CS [12–14]. Systemic inflammation, as indicated by elevated plasma concentrations of inflammatory mediators such as C-reactive protein (CRP), is frequently observed among patients with CS and considered a major pathophysiologic mechanism contributing to worsening shock and multi-organ injury [3, 15–19]. In patients with sepsis induced CS treatment with MCS might even be associated with a higher survival as indicated by a retrospective analysis [20]. We, therefore, hypothesized that systemic inflammation may be a key factor contributing to the downward spiral of CS characterized by aggravated tissue hypoperfusion. And in turn, this patient group in particular may benefit from MCS restoring blood flow and providing improved tissue perfusion.

Hence, this study is aimed to explore the association of systemic inflammation with shock severity, 30-day in-hospital mortality, and therapy response in patients with CS. Improving its understanding might facilitate the development of successful treatment strategies and conduction of clinical trials in this field.

## Methods

### Study design

This is a retrospective analysis in the previously reported [4] monocentric Hamburg Cardiogenic Shock registry of the University Heart and Vascular Center Hamburg. The registry includes patients aged 18 years or older suffering from CS between 2009 and 2019. To identify eligible patients, patient records were electronically scanned for patients with CS according to ICD-10 coding (R.57). All records were manually validated by a physician to confirm CS as the primary cause of the shock leading to hospital admission. Patients with a primarily septic shock were not included. This was verified by a manual chart review (documented clinical and laboratory data) of all included cases. Baseline data, comorbidities, and treatment data were collected in a dedicated database. Patients were followed up until death or discharge from hospital.

The aim of this study was to explore the association of systemic inflammation with shock severity, 30-day in-hospital mortality, and therapy response in patients with CS. As surrogate parameter for systemic inflammation plasma CRP concentrations on admission were used, so that patients without available data for plasma CRP concentrations were excluded from this study. The severity of CS was defined based on the published SCAI shock classification [21] as well as on the use and number of vasopressors. Also, as surrogate for shock duration lactate measurements on admission were evaluated in relation to plasma CRP concentrations. Therapy response was assessed as survival with use of MCS (Veno-arterial extracorporeal membrane oxygenation therapy (VA-ECMO) and/or Impella^®^).

This study conforms with the principles outlined in the Declarations of Helsinki, and was approved by our local ethics committee (registration code PV5607). Due to the retrospective study design all patient data were anonymized, hence the need to give informed consent was waived.

### Statistical analyses

Baseline characteristics were generated for the whole cohort, as well as for the subgroups with high vs. low plasma CRP concentrations, dichotomized by CRP median of the study cohort. Continuous variables are expressed as medians with interquartile range and compared using the Mann–Whitney *U* test. Categorical variables are shown as absolute numbers and percentages and compared using the chi^2^ test. *p*-values < 0.05 were considered significant.

To evaluate the association between plasma CRP concentrations and SCAI stage linear and logistic regression models were fitted, using SCAI B as reference category. Plasma CRP concentrations were first analyzed as continuous variable per 50 mg/l CRP increase (linear regression) and subsequently as dichotomous variable using the CRP median (logistic regression). To examine the relationship between plasma CRP concentrations and lactate concentrations (as a surrogate for CS duration) as well as the use and number of vasopressors linear and logistic regression models were chosen correspondingly.

To assess the relative risk of 30-day in-hospital mortality in association with plasma CRP concentrations, Kaplan–Meier method was applied and Cox regression models were fitted, again using plasma CRP as a continuous and/or dichotomous variable as stated above. Groups were compared using the log-rank test. To evaluate the relative risk of 30-day in-hospital mortality in association with plasma CRP concentrations within each SCAI stage subgroup analyses including only patients with SCAI stage B and C combined, D and E were performed.

To evaluate the interaction between plasma CRP concentrations and MCS use regarding the relative risk of 30-day in-hospital mortality an interaction term between plasma CRP concentrations and MCS use was fitted into the above-described cox regression models. Analyses were performed for MCS use in general and each device separately. To account for differences in patient, presentation and shock-specific characteristics in patients with high vs. low plasma CRP concentrations, all analyses were adjusted for the following potential confounders based on clinical knowledge and prior studies [22]: age, sex, AMI, lactate, pH, cardiopulmonary resuscitation (CPR), patient intubated due to index event and SCAI stage (if not the endpoint). For the multivariable adjusted regression models, only patients with data on all confounders were considered (e.g., complete case analysis).

All analyses were performed using the software R version 4.0.3.

## Results

### Baseline characteristics

The data set contains information from 1338 patients with CS unselected for its etiology. 201 patients without information about plasma CRP concentrations were excluded, so that 1116 patients remained for this analysis. Baseline characteristics of the overall cohort and of those with high and low CRP concentrations (split by median CRP) are displayed in Table 1.

**Table 1:** Baseline characteristics of the overall cohort as well as stratified by high vs. low CRP concentration (CRP > vs. ≤ median)

	All (*N* = 1116)	CRP ≤ median (*N* = 569)	CRP > median (*N* = 547)	*p*-value	Missing values in %
*Baseline characteristics*					
Age (years)	70.0 (58.0, 78.6)	68.0 (55.0, 78.0)	71.0 (61.0, 79.0)	< 0.001	0.7
Male No. (%)	795 (71.3)	419 (73.8)	376 (68.7)	0.074	0.1
*Cardiovascular risk factors*					
Arterial hypertension No. (%	535 (51.3)	255 (48.8)	280 (53.8)	0.11	6.5
Diabetes mellitus No. (%)	288 (27.5)	133 (25.4)	155 (29.6)	0.15	6.1
CKD No. (%)	204 (19.3)	73 (13.8)	131 (24.8)	< 0.001	5.4
Prior CABG No. (%)	113 (10.6)	59 (11.0)	54 (10.2)	0.71	4.6
*Presentation*					
CPR No. (%)	648 (58.3)	383 (67.4)	265 (48.7)	< 0.001	0.4
Duration of CPR (per 10 min)	14 (1.5)	5 (1.0)	9 (2.0)	< 0.001	17.4
Patient intubated due to index event No. (%)	781 (70.9)	422 (75.0)	359 (66.7)	0.0033	1.3
*Cause of CS*					
Acute myocardial Infarction No. (%)	530 (47.7)	290 (51.1)	240 (44.0)	0.021	0.4
*Ejection fraction*					
Preserved No. (%)	143 (16.0)	82 (17.7)	61 (14.2)	0.18	19.8
Mid-ranged No. (%)	139 (15.5)	93 (20.0)	46 (10.7)	< 0.001	19.8
Reduced No. (%)	613 (68.5)	289 (62.3)	324 (75.2)	< 0.001	19.8
*SCAI-class*					
B No. (%)	45 (4.1)	16 (2.9)	29 (5.4)	0.047	1.9
C No. (%)	569 (52.0)	293 (52.3)	276 (51.6)	0.86	1.9
D No. (%)	254 (23.2)	102 (18.2)	152 (28.4)	< 0.001	1.9
E No. (%)	227 (20.7)	149 (26.6)	78 (14.6)	< 0.001	1.9
*Hemodynamics*					
Systolic blood pressure (mmHg)	101.0 (83.0, 125.0)	104 (83.0, 130.0)	100.0 (83.0, 120.0)	0.78	10.9
Heart rate (bpm)	89.0 (71.0, 110.0)	86.0 (68.0, 107.0)	92.0 (75.0, 114.0)	< 0.001	10.2
*Laboratory*					
pH	7.3 (7.1, 7.4)	7.2 (7.1, 7.4)	7.3 (7.2, 7.4)	< 0.001	7.9
Lactate (1st measurement) (mmol/L)	4.6 (2.2, 9.5)	5.3 (2.5, 10.0)	4.1 (2.0, 8.7)	0.0088	9.8
calculated GFR (ml/min)	41.6 (26.9, 57.9)	47.2 (35.0, 62.8)	32.9 (20.3, 51.3)	< 0.001	1.3
CRP (mg/l)	17.0 (5.0, 71.0)	5.0 (5.0, 8.0)	72.0 (38.0, 126.0)	< 0.001	–
*Therapy*					
MCS use No. (%)	312 (28.2)	180 (31.6)	134 (24.6)	0.011	0.3
VA-ECMO No. (%)	257 (23.1)	151 (26.5)	106 (19.5)	0.0065	0.3
Impella^®^ No. (%)	158 (14.2)	89 (15.6)	69 (12.7)	0.18	0.3

Values of categorical variables are displayed as absolute numbers with the relative frequency in per cent and compared using the chi^2^ test. Values of continues variables are displayed with their median and interquartile range and compared using Mann–Whitney *U* test. Missing values of the overall cohort are given in per cent

*CDK* chronic kidney disease, *CABG* coronary artery bypass graft, *CPR* cardio-pulmonary resuscitation, *SCAI*-*class* shock stage classification by the Society for Cardiovascular Angiography and Interventions, *GFR* glomerular filtration rate, *CRP* C-reactive protein, *MCS* *use* use of mechanical circulatory support device, *VA*-*ECMO* veno-arterial extracorporeal membrane oxygenation therapy

Median age of all patients was 70 (58–78.6) years and 795 (71.3%) were male. Almost half of the patients presented with CS due to AMI (*n* = 530, 47.7%) and prior cardiac arrest (CA) (*n* = 648, 58.3%). Median pH was 7.3 (7.1–7.4), lactate 4.6 (2.2–9.5) mmol/l and GFR 41.6 (26.9–57.9) ml/min. The overall distribution of SCAI stages was B: 45 patients (4.1%), C: 569 patients (52%), D: 254 patients (23.2%) and E: 227 patients (20.7%). MCS was used in 314 patients (28.2%), including VA-ECMO in 257 patients (23.1%) and Impella^®^ in 158 patients (14.2%). 101 patients (9.1%) were treated with both VA-ECMO and Impella^®^. The median plasma CRP concentration in the study population was 17 (5.0–71.0) mg/l. Patients with CRP > median were slightly older (CRP > median: 71 (61–79) years; CRP ≤ median: 68 (55–78) years) and less often male (CRP > median: *n* = 376, 68.7%; CRP ≤ median: *n* = 419, 73.8%). The prevalence of CS due to AMI and CA was slightly reduced in the group of patients with CRP > median (AMI: CRP > median: *n* = 240, 44.0%; CRP ≤ median: *n* = 290, 51.1% and CA: CRP > median: *n* = 265, 48.7%; CRP ≤ median: *n* = 383, 67.4%). The use of MCS was slightly less observed in patients with CRP > median (CRP > median: MCS: *n* = 134, 24.6% with VA-ECMO: *n* = 106, 19.5% and Impella^®^: *n* = 69, 12.7%; CRP ≤ median: MCS: *n* = 180, 31.6% with VA-ECMO: *n* = 151, 26.5% and Impella^®^: *n* = 89, 15.6%).

### Systemic inflammation and shock severity

**Fig. 1:**
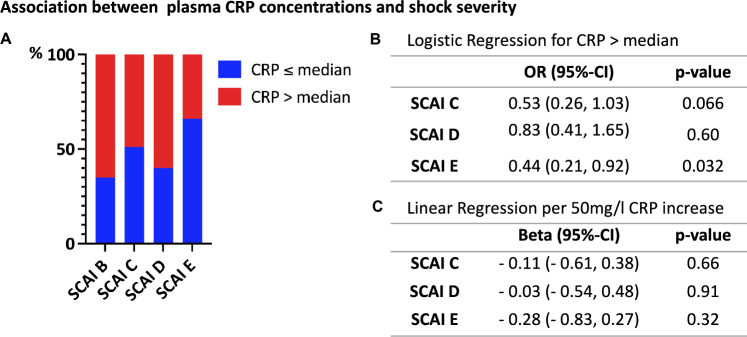
Association between plasma CRP concentrations and shock severity (SCAI stage) in patients with cardiogenic shock. **A** Percental distribution of CRP > vs. ≤ median per SCAI stage. **B** Logistic regression with odds ratio (OR) for CRP > median. **C** Linear regression per 50 mg/l CRP increase. CRP: C-reactive protein. SCAI-stage: shock stage classification by the Society for Cardiovascular Angiography and Interventions. *95%-CI* 95% confidence interval. All regression models adjusted for age, sex, acute myocardial infarction, lactate, pH, cardiopulmonary resuscitation (CPR) and patient intubated due to index event

As the SCAI shock stage (shock severity) increased, plasma CRP concentrations appeared to decrease but after adjustment for relevant confounders, there was no consistent association between plasma CRP concentrations and shock severity, irrespective of being considered as continuous [beta (*β*) per 50 ml/l CRP increase with reference SCAI B: SCAI C (*β* − 0.11, 95% confidence interval (CI): − 0.61, 0.38; *p* = 0.66), SCAI D (*β* − 0.03, 95%-CI − 0.54, 0.48; *p* = 0.91); SCAI E (*β* − 0.28, 95%-CI − 0.83, 0.27; *p* = 0.32)] or dichotomized variable [Odds Ratio (OR) for CRP > median: SCAI C (OR 0.53, 95%-CI 0.26, 1.03; *p* = 0.066), SCAI D (OR 0.83, 95%-CI 0.41, 1.65; *p* = 0.6); SCAI E (OR 0.44, 95%-CI 0.21, 0.92; *p* = 0.032)] as shown in Fig. 1.

Higher CRP concentrations were associated with higher lactate concentrations (i.e., presumably a longer duration of CS). When considered as dichotomized variable this association persisted even after adjustment for relevant confounders including SCAI stage (OR for CRP > median: 1.06, 95%-CI 1.02, 1.10; *p* = 0.003). When considered as continuous variable this association was only statistically significant before but not after adjustment (*β* per 50 ml/l CRP increase: 0.01, 95%-CI − 0.02, 0.04; *p* = 0.47).

Higher CRP concentrations were partly associated with use of vasopressors. Detailed results are given in Appendix Table 2.

### Systemic inflammation and 30-day mortality

**Fig. 2:**
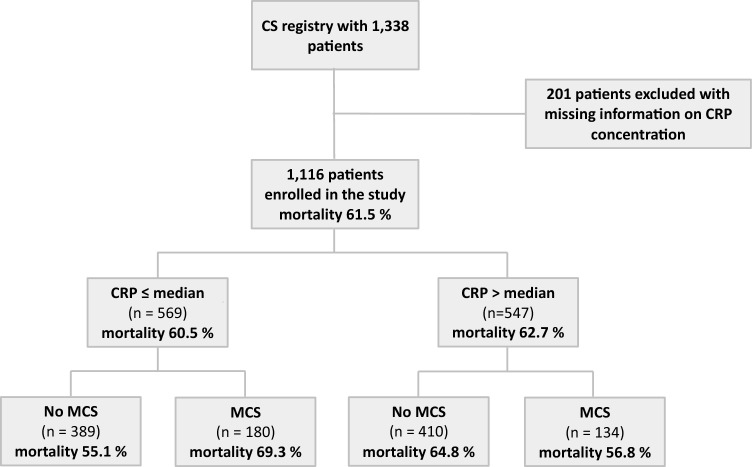
30-day mortality of the overall cohort and according to CRP concentration and treatment response (MCS use) in patients with cardiogenic shock. *CS* cardiogenic shock, *CRP* C-reactive protein, *MCS use* use of mechanical circulatory support device

Patients were followed-up for a median in-hospital follow-up time of 25 (95%-CI 23, 28) days. A total of 626 patients died within the 30-day follow up, yielding a 30-day in-hospital mortality rate of 61.47% (95%-CI 58.09, 64.58%). Patients with higher CRP concentrations had a higher 30-day in-hospital mortality [62.68% (95%-CI 57.97, 66.86%)] than patients with lower CRP concentrations [60.49% (95%-CI 55.41, 65.0%)] (Fig. 2). Kaplan–Meier survival curves are displayed in Appendix Fig. 4.

**Fig. 3:**
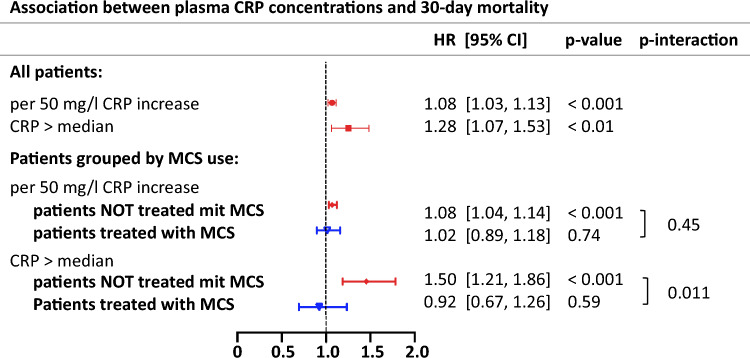
Association between plasma CRP concentrations, 30-day mortality and therapy response (MCS use) in patients with cardiogenic shock. *CRP* C-reactive protein, *MCS use* use of mechanical circulatory support device, *HR* hazard ratio per 50 mg/l CRP increase and CRP > vs. ≤ median, *95%-CI* 95% confidence interval. Cox regression models adjusted for age, sex, acute myocardial infarction, lactate, pH, cardiopulmonary resuscitation (CPR), patient intubated due to index event and SCAI-stage

After adjustment for potential confounders, higher plasma CRP concentrations were associated with a higher risk of 30-day in-hospital mortality (Fig. 3): when considered as a continuous variable we found an 8% relative risk increase per 50 mg/l increase in plasma CRP, range 3–13% (Hazard Ratio (HR) 1.08, 95%-CI 1.03, 1.13; *p* < 0.001, when considered as a dichotomized variable the observed relative risk increase was 28% in the group of CRP > median, range 7–53% (HR 1.28, 95%-CI 1.07, 1.53; *p* = 0.0065). Corresponding subgroup analyses within each SCAI stage are given in Appendix Table 3*.*

### Systemic inflammation and therapy response

Patients with higher CRP concentrations had a lower 30-day mortality when treated with MCS [56.78% (95%-CI 47.28, 64.57%)] compared to those not treated with MCS [64.81% (95%-CI 59.11, 69.72%)]. Conversely, patients with lower CRP concentrations had a higher 30-day in-hospital mortality when treated with MCS [69.3% (95%-CI 61.05, 75.8%) vs. 55.13% (95%-CI 48.64, 60.8%)] (Fig. 2). For Kaplan–Meier curves see Appendix Fig. 5*.* In the adjusted analysis, elevated plasma CRP concentrations appeared to only be associated with a higher mortality risk in patients not treated with MCS compared to those treated with MCS, although the interaction was only statistically significant if plasma CRP concentration was considered as a dichotomized variable: relative risk increase of 50% with plasma CRP concentrations > median in patients without MCS use (HR 1.50, 95%-CI 1.21, 1.86; *p* < 0.001) vs. no significant risk increase with plasma CRP concentrations > median in patients with MCS use (HR 0.92, 95%-CI 0.67, 1.26; *p* = 0.59; *p*-interaction = 0.01; Fig. 3). Results looking at each device separately (VA-ECMO, Impella^®^, ECMELLA) replicated these findings and are given in Appendix Table 4*.*

## Discussion

The key findings of our analysis aiming to investigate the role of systemic inflammation in patients with CS were, firstly, plasma CRP concentrations seem to be equally distributed in patients with higher vs. lower shock severity. Secondly, even after adjustment for variables representing patient, clinical and shock-specific characteristics, plasma CRP concentration was a strong predictor of 30-day in-hospital mortality risk. And thirdly, higher plasma CRP concentrations were only associated with higher mortality in patients not treated with MCS, but not in those treated with MCS, indicating that MCS use might mitigate the hazardous impact of systemic inflammation in CS.

CS is increasingly recognized as heterogenous in underlying disease mechanisms, severity and response to therapies [4, 14, 23, 24]. The pathophysiology of CS includes more than only the reduction of cardiac output but instead also involves factors such as microvascular disturbances and systemic inflammation, which in combination contribute to multi-organ failure [3, 15, 17]. While this systemic inflammation can be caused by bacterial infection, a sterile systemic inflammation, also known as systemic inflammatory response (SIRS), can especially be triggered by various other factors [15], most of which are common in CS, such as tissue hypoxia/damage [25, 26], myocardial pressure overload [27] and activation of the sympathetic system [28]. The involvement of systemic inflammation in CS is highlighted by biomarker studies reporting an increase in plasma concentrations of CRP and inflammatory cytokines such as IL-6 and TNFα in patients with CS [16, 29], being correlated with the severity of CS and linked to a poor prognosis [18, 19]. A recent study suggested SIRS was increasingly prevalent as the severity of CS increased and associated with higher in hospital mortality [13]; moreover, IL-6 was found to be an independent predictor of early mortality in patients with AMI-CS [18]. Also, CRP has been found to predict cardiovascular mortality in asymptomatic adults and patients with acute coronary syndrome (ACS) [30, 31].

Contrary to these findings and what one might expect in our study population plasma CRP concentrations were not correlated with shock severity based on the SCAI classification. One explanation of this discrepancy could be a more detailed adjustment within the scope of our analysis, especially as the statistical association dissolved with increasing adjustment variables. A further explanation could be, that these prior studies have focused primarily on patients with AMI-CS, whereas our study population included all etiology CS. Also, higher CRP concentrations showed a strong association with higher lactate concentrations which are directly linked to the extent of tissue hypoperfusion and therefore shock duration. We hypothesize, that a slow but progressive onset of CS leaves more time for the patients to develop higher CRP concentrations compared to patients with a highly acute and severe onset as commonly seen in AMI and CA cases. This also explains the higher rate of CPR, AMI and ventilation in the group with lower CRP levels. The association between CRP and lactate persisted despite adjustment including SCAI stage indicating systemic inflammation might be a separate and independent component affecting patients across all different SCAI stages.

Furthermore, this study identified a strong association between plasma CRP concentrations and 30-day in-hospital mortality in patients with CS. This effect was present even when accounting for the established predictors of mortality such as lactate [32] and CA [33] as well as severity of CS, using the SCAI shock classification [4]; and is consistent with above mentioned prior published literature. While the reduction of cardiac output undoubtedly represents the initial driving factor of CS, maladaptive compensatory mechanisms in dissociation from the initial cardiac damage, including a systemic inflammatory response, seem to be central in fueling the endless downward spiral leading to refractory CS. Therefore, these data further support the idea that the presence of systemic inflammation may be a clinically useful tool to identify higher risk patients and provide additional risk stratification within the SCAI staging criteria. One key advantage being, CRP is an easily accessible and widely used laboratory parameter in daily clinical practice that could serve as surrogate parameter for systemic inflammation.

In addition to prognostication, it is important to evaluate whether the benefit of select therapies differs based on the presence of systemic inflammation. In particular regarding the use of MCS in patients with systemic inflammation it has been questioned if restoring macro-circulatory hemodynamics is sufficient to halt the downward spiral of CS and is effective for raising blood pressure and allowing vasopressors to be weaned in a mixed cardiogenic-vasodilatory shock phenotype with inadequate peripheral vascular tone [23]; also complicated by the fact that MCS provide additional triggers for a dysregulated inflammatory response [34, 35]. Furthermore, adverse events associated with MCS use are frequent calling for careful patient selection [9, 36].

Data regarding CS management, especially MCS and systemic inflammatory response remain scarce. A previous study showed a reduction in IL-6 after MCS use and its association with survivorship [37], contrary to other findings reporting a reduction in IL-6 after MCS use regardless of the outcome [38]. In this study, we could show that elevated plasma CRP concentrations appeared to only be associated with higher mortality in patients not treated with MCS, but not in those treated with MCS. This may be affected by selection bias, as only patients with perceived benefit were chosen for MCS. Also, the argument could be made that the use of MCS reflects the severity of CS. In more severely ill patients not responding to treatment other predictors of mortality could be more important than CRP concentrations. However, the association remained after adjusting for severity of CS and other predictors of mortality. Also, the results of CRP measurements were not yet available when the decision for or against MCS was made.

Therefore, we hypothesize systemic inflammation may play a central role contributing to the downward spiral of CS. MCS use might mitigate this negative impact of systemic inflammation potentially by providing sufficient end-organ perfusion with reduction of metabolic derangements, inflammatory cytokines and oxidative stress which could alleviate further microcirculatory dysfunction caused by CS. Hence the argument could be made that aside from a causal and timely treatment of the underlying condition which is the utmost important element, a promptly use of MCS in patients with systemic inflammation is also important to contain the downward spiral of CS. The presence or absence of systemic inflammation, measured by CRP concentrations or other inflammatory markers, may one day play a role in therapeutic decision making regarding MCS in CS. In light of the recently published ECLS-shock trial [39] and associated metanalysis [40] by Thiele et al. it is important to recognize the calling for a careful review of the indication for VA-ECMO treatment as it demonstrated no benefit in 30-day mortality for AMI-CS patients treated vs. not treated with VA-ECMO. It would be interesting if our hypothesis could be validated in a substudy of the ECLS-shock trial looking at the mortality of the subpopulation with increased systemic inflammation.

### Limitations

One major limitation of this study is its retrospective and monocentric study design which includes several factors: Firstly, the possibility of a selection bias as patients were identified via ICD-10 coding. This might have led to the inclusion of misdiagnosed patients who did not actually suffer from CS but other causes of shock. However, this is rather unlikely as a careful manual chart review by a physician was performed in all cases. Secondly, not all aspects of the SCAI classification were available (e.g., specific biomarkers or invasive hemodynamics) potentially leading to a misclassification regarding SCAI shock stage. Thirdly, the potential of missing data and unmeasured or unknown confounders preclude from establishing causal relationships.

In addition, we acknowledge systemic inflammation is the result of different inflammatory mechanisms and mediators and may not be represented holistically by the exclusive analysis of plasma CRP concentrations. Plasma CRP is a sensitive yet non-specific biomarker of systemic inflammation and induced under various conditions including infection, trauma and other inflammatory states such as autoimmune disease. This could lead to difficulties regarding a clear interpretation, especially in patients with additional infections where CRP concentrations may affect therapy and prognosis.

Our study merely describes associations and is not designed to investigate whether systemic inflammation plays a causal role in shock severity, 30-day mortality and therapy response. Larger multicentre prospective studies are necessary to confirm our findings and animal studies are necessary to establish causation between systemic inflammation and CS.

### Conclusion

In patients with CS increased concentrations of plasma CRP are associated with a higher 30-day mortality regardless of shock severity; which is consistent with prior studies associating increased inflammatory markers with a poor prognosis. Furthermore, our results indicate that MCS use might mitigate this negative impact of systemic inflammation. Overall, plasma CRP is an easily accessible marker of systemic inflammation that could serve as independent predictor of outcomes in patients with CS. Also, CRP could potentially be considered for therapeutic decision making regarding the use of MCS in CS and calls for further research on this topic.

## Figures and Tables

**Fig. 4:**
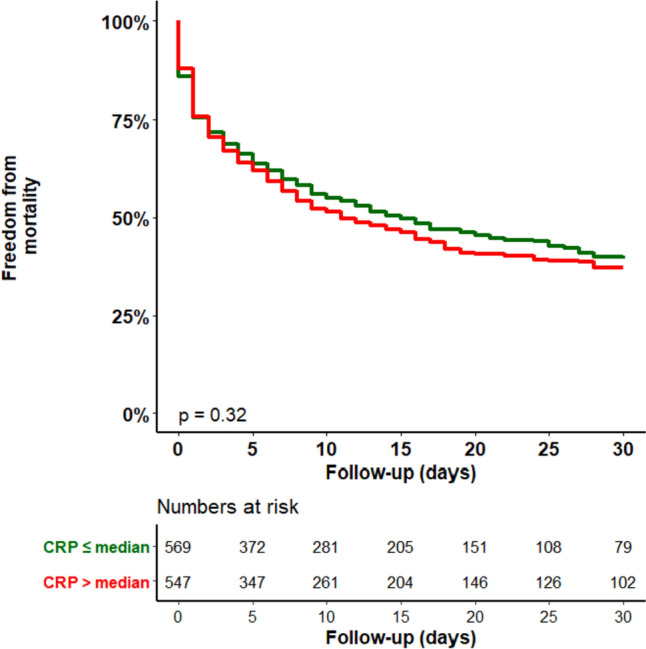
Kaplan–Meier curves for elevated vs. non-elevated CRP concentration in patients with CS

**Fig. 5:**
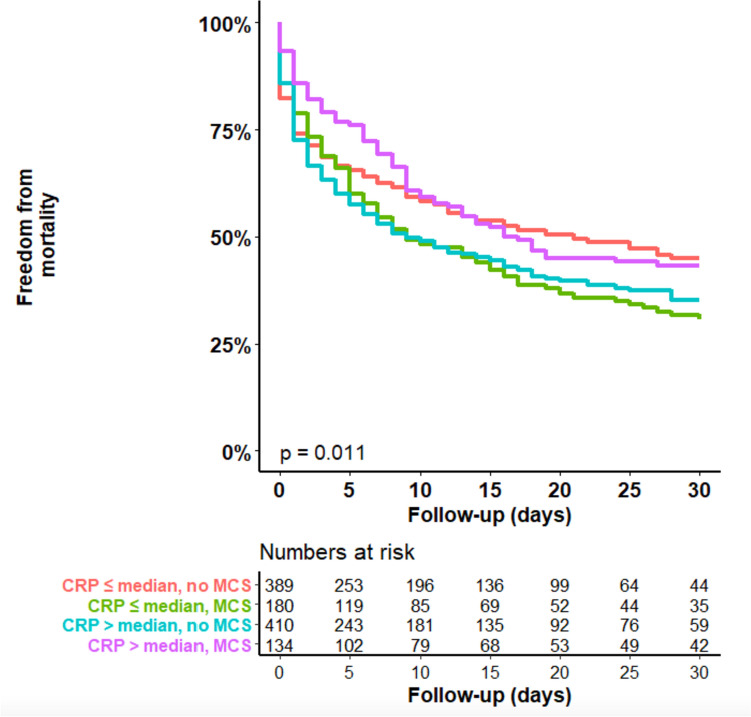
Kaplan–Meier-curves for treatment response in CS patients with elevated and non-elevated CRP concentration

## Appendices

See Tables 2, 3, 4 and Figs. 4 and 5.

**Table 2:** Association between plasma CRP concentration and use of vasopressors in patients with CS

	*β*/OR	95%-CI	*p*-value
*Inotropes (yes/no)*			
Per 50 mg/l CRP increase	*β* 0.33	1.05, 1.61	0.023
For CRP > median	OR 1.40	0.99, 1.99	0.057
*Inotropes (No.)*			
Per 50 mg/l CRP increase			
Inotropes (1)	*β* 0.33	0.04, 0.62	0.024
Inotropes (≥ 2)	*β* 0.28	− 0.44, 0.99	0.45
For CRP > median			
Inotropes (1)	OR 1.45	1.01, 2.07	0.042
Inotropes (≥ 2)	OR 1.01	0.42, 2.50	0.98

**Table 3:** Association between CRP concentration and 30-day mortality within each SCAI stage

	HR	95%-CI	*p*-value
*SCAI B + C*			
Per 50 mg/l CRP increase	1.08	1.02, 1.14	< 0.01
For CRP > median	1.40	1.07, 1.83	0.015
*SCAI D*			
Per 50 mg/l CRP increase	1.06	0.95, 1.19	0.28
For CRP > median	1.21	0.85, 1.70	0.29
*SCAI E*			
Per 50 mg/l CRP increase	1.16	1.00, 1.34	0.051
For CRP > median	1.19	0.86, 1.67	0.30

**Table 4:** Association between CRP concentration, 30-day mortality and therapy response to each MCS device separately

	HR	95%-CI	*p*-value	*p*-interaction
*VA-ECMO*				
Per 50 mg/l CRP increase				
Patients NOT treated with VA-ECMO	1.09	1.04, 1.14	< 0.001	0.31
Patients treated with VA-ECMO	1.00	0.085, 1.19	0.067	
For CRP > median				
Patients NOT treated with VA-ECMO	1.45	1.18, 1.78	< 0.001	0.02
Patients treated with VA-ECMO	0.91	0.64, 1.29	0.59	
*Impella* ^*®*^				
Per 50 mg/l CRP increase				
Patients NOT treated with Impella^®^	1.08	1.03, 1.13	< 0.001	0.71
Patients treated with Impella^®^	1.04	0.84, 1.28	0.73	
For CRP > median				
Patients NOT treated with Impella^®^	1.41	1.16, 1.71	< 0.001	0.04
Patients treated with Impella^®^	0.84	0.54, 1.31	0.44	
*ECMELLA*				
Per 50 mg/l CRP increase				
Patients NOT treated with ECMELLA	1.08	1.04, 1.13	< 0.001	0.41
Patients treated with ECMELLA	0.96	0.73, 1.27	0.79	
For CRP > median				
Patients NOT treated with ECMELLA	1.38	1.14, 1.67	< 0.001	0.05
Patients treated with ECMELLA	0.77	0.44, 1.34	0.35	

## Abbreviations

ACS: Acute coronary syndrome
AMI: Acute myocardial infarction
CA: Cardiac arrest
CRP: C-reactive protein
CS: Cardiogenic shock
MCS: Mechanical circulatory support
PCI: Percutaneous coronary intervention
SCAI: Society for Cardiovascular Angiography and Intervention
SIRS: Systemic inflammatory response
VA-ECMO: Veno-arterial extracorporeal membrane oxygenation therapy
